# Stability of hospital quality indicators over time: A multi-year observational study of German hospital data

**DOI:** 10.1371/journal.pone.0293723

**Published:** 2023-11-07

**Authors:** Nils Patrick Kollmann, Benedikt Langenberger, Reinhard Busse, Christoph Pross

**Affiliations:** 1 Helios IT Service GmbH, Berlin, Germany; 2 Department of Health Care Management, Berlin University of Technology, Berlin, Germany; Center for Primary Care and Public Health: Unisante, SWITZERLAND

## Abstract

**Background:**

Retrospective hospital quality indicators can only be useful if they are trustworthy signals of current or future quality. Despite extensive longitudinal quality indicator data and many hospital quality public reporting initiatives, research on quality indicator stability over time is scarce and skepticism about their usefulness widespread.

**Objective:**

Based on aggregated, widely available hospital-level quality indicators, this paper sought to determine whether quality indicators are stable over time. Implications for health policy were drawn and the limited methodological foundation for stability assessments of hospital-level quality indicators enhanced.

**Methods:**

Two longitudinal datasets (self-reported and routine data), including all hospitals in Germany and covering the period from 2004 to 2017, were analysed. A logistic regression using Generalized Estimating Equations, a time-dependent, graphic quintile representation of risk-adjusted rates and Spearman’s rank correlation coefficient were used.

**Results:**

For a total of eight German quality indicators significant stability over time was demonstrated. The probability of remaining in the best quality cluster in the future across all hospitals reached from 46.9% (CI: 42.4–51.6%) for hip replacement reoperations to 80.4% (CI: 76.4–83.8%) for decubitus. Furthermore, graphical descriptive analysis showed that the difference in adverse event rates for the 20% top performing compared to the 20% worst performing hospitals in the two following years is on average between 30% for stroke and AMI and 79% for decubitus. Stability over time has been shown to vary strongly between indicators and treatment areas.

**Conclusion:**

Quality indicators were found to have sufficient stability over time for public reporting. Potentially, increasing case volumes per hospital, centralisation of medical services and minimum-quantity regulations may lead to more stable and reliable quality of care indicators. Finally, more robust policy interventions such as outcome-based payment, should only be applied to outcome indicators with a higher level of stability over time. This should be subject to future research.

## Introduction

Despite increasing investments and continuous reform efforts, studies repeatedly demonstrate significant patient safety issues and variations in hospital quality in the health systems of Europe and the US [1–3]. For many treatment areas, there is wide variation in quality among individual hospitals within national health systems [4–7]. For example, Pross *et al*. [8] found that German hospitals in the fifth (worst) quality quintile had three to twelve times worse outcomes than hospitals in the best quintile across six risk-adjusted mortality and reintervention rates in surgical and non-surgical treatment areas.

Reducing unwarranted variation in care quality and reliably identifying conspicuously good and bad providers are increasingly important aspects of health policy and care improvement initiatives [9]. Valid and fair quality measurement is critical for benchmarking and best practice development. Reliable publicly reported care quality information can enable patients to take an active role in deciding the best healthcare provider for their needs [9, 10].

Retrospective quality indicators are the basis for quality assurance and all associated care quality improvement initiatives [11–14]. Quality indicators must be methodologically and statistically sound to ensure information value [15]. However, knowledge of historical quality based on retrospective quality indicators does not necessarily provide information about the current or future quality of a hospital [16–18]. In the field of health policy, a quality indicator is only actionable, meaning fit for purpose and fit for use, if its results among other relevant criteria (e.g. from RAND/UCLA Appropriateness Method or QUALIFY catalogs) are stable over time as decisions for the future are made on the basis of historical data [19–23].

Yet current research on the usefulness of retrospective quality for present or future hospital choice has several gaps. *First*, several studies investigate the reliability of hospital rankings (rankability) and stability of the hospital effects based on one to three year’s indicator results; [24–34] however, analyses on the stability of these rankings over a longer period are limited. *Second*, the geographic coverage of existing studies is limited. Studies focus mainly on the US or the Netherlands, as of yet there are no studies from Germany. *Third*, current literature mainly examines rankability and is limited with regards to stability over time [28, 32, 43, 44]. *Fourth*, while the studies that do exist are consistent on the positive correlation of hospital rankability and event rate/case volume, [28] they provide mixed conclusions on the usefulness of the examined outcome indicators. Several find good or at least sufficient stability or rankability over time [16, 17, 25, 35–37]. Others describe strong fluctuations and thus low stability for some of the same and other treatment areas [18, 25, 27, 30, 33, 34, 38, 39]. *Fifth*, a mix of different methods is used, with no consensus on how to assess the stability of quality indicators. Finally, the used indicators were often reliability-adjusted as part of the evaluations, though most published indicator results are not, meaning the study findings cannot be used without restrictions to make statements on the indicators used in practice by patients, clinicians and policy makers.

Using hospital-level, aggregated indicator data extracted from Germany’s Mandatory National Quality Monitoring System system [40, 41] on eight different surgical and non-surgical quality indicators across seven treatment areas, this study aimed to identify the stability over time and information value for the current and future quality of hospitals. Furthermore, this study expands the limited previous GEE application to hospital quality indicators with a GEE model using more widely available aggregated, non-reliability adjusted quality indicators. Lastly, tangible lessons for health policy related to quality of care transparency were concluded and the suitability of the applied method in terms of stability in the field of outcome quality indicators in healthcare was confirmed.

## Methods

### Data

Two longitudinal datasets with risk-adjusted, hospital-level outcome O/E-ratios (observed/expected rate) were used for the analysis of eight quality indicators. Indicators were selected based on available data years, comparability over time and beneficial statistical characteristics (e.g. large number of hospitals, high case volume per hospital) from the following two sources:

(1) Mandatory National Quality Monitoring System (six indicators): The Institute for Quality Assurance and Transparency in Healthcare (IQTIG) and prior the aQua-institute use self-reported hospital data to calculate hospital-level O/E-ratios. Annual risk-adjustment by means of logistic regression that include patient-specific risk-factors such as age, gender, and co-morbidities is performed by each institute (for variables and regression weights see the methodological S1 Appendix or risk statistics of the institutes) [42–45]. Non-personal, hospital level data for the full hospital census (~1,800 hospitals) is made publicly available by the *Gemeinsamer Bundesausschuss* (G-BA) and the *Weisse Liste*. For purposes of this study, indicator results (O/E ratios) from 2006 to 2017 (see Table 1) were used. Due to missing data for data protection reasons in Rhineland-Palatinate in 2012 and in parts of North Rhine-Westphalia in 2016, hospitals from these regions were excluded.

**Table 1:** Indicator description with overview on the data sources, treatment area, measure period and data years.

Data source	Indicator abbreviation	Treatment area	Indicator description	Measure period	Data included	Data years													
						04	05	06	07	08	09	10	11	12	13	14	15	16	17
Mandatory National Quality Monitoring; self-reported by hospitals; aggregated on hospital-level; publicly available ^1^	PNEU	Community acquired pneumonia	Ratio of the observed to the expected (O/E) rate of deaths, risk-adjusted (SMR)	Inpatient stay	Total population, GER excl. Rhineland-Palatinate and Northern North Rhine-Westphalia									*	*	*	*	*	*
	DECU	Decubitus ulcer	Ratio of the observed to the expected rate (O/E) in patients with at least one decubitus ulcer acquired in hospital (without decubital ulcer grade/category 1), risk-adjusted	Inpatient stay	Total population, age > 20 years, hospitals > 20 calculated cases in risk statistics, GER excl. Northern North Rhine-Westphalia											*	*	*	
	CHOLEC	Cholecystectomy	Ratio of the observed to the expected rate (O/E) of reinterventions due to complications, risk-adjusted	Inpatient stay	Total population, age > 20 years, GER excl. Rhineland-Palatinate									*	*	*			
	HIPFR	Hip fracture repair	Ratio of the observed to the expected (O/E) rate of deaths, risk-adjusted (SMR)	Inpatient stay	Total population, births from the 24^th^ pregnancy week, GER excl. Northern North Rhine-Westphalia									*	*	*			
	HIPREPDI	Hip replacement	Ratio of the observed to the expected rate (O/E) of implant malpositions, dislocations or fractures, risk-adjusted	Inpatient stay	Total population, GER excl. Rhineland-Palatinate and Northern North Rhine-Westphalia									*	*	*			
	HIPREPRE		Ratio of the observed to the expected rate (O/E) of reoperations due to complications, risk-adjusted	Inpatient stay	Total population, age > 20 years, ASA 1 to 3, GER excl. Rhineland-Palatinate									*	*	*			
Administrative Data of the AOK sickness funds; calculated centrally by WIdO and aggregated on hospital-level; not publicly available ^2^	STROKE	Stroke	Ratio of the observed to the expected (O/E) rate of deaths, risk-adjusted (SMR)	30 days from hospital admission	AOK insured age > 30 years, GER	*	*	*	*	*	*	*	*	*	*	*			
	AMI	Acute myocardial infarction (AMI)	Ratio of the observed to the expected (O/E) rate of deaths, risk-adjusted (SMR)	30 days from hospital admission	AOK insured age > 30 years, GER	*	*	*	*	*	*	*	*	*	*	*			

Notes:

^1^ Hospital quality report cards are made publicly accessible by the Gemeinsamer Bundesausschuss (G-BA) and the Weisse Liste within the Mandatory National Quality Monitoring Program. Due to missing data for data protection reasons in Rhineland-Palatinate in 2012 and in parts of North Rhine-Westphalia in 2016, hospitals from these areas were excluded.

^2^ QSR indicators are based on routine data (up to one year follow up) for inpatient stay of AOK-insured patients [46]. AOK is the biggest health insurance company in Germany, with around 26.8 million insured persons and more than 36% of the statutory health insurance market in 2019 [47]. Indicators are partially publicly accessible. QSR indicators were aggregated at hospital level and matched with the Mandatory National Quality Monitoring data using unique hospital IDs and address data.

(2) Administrative Data of the AOK Sickness Funds (QSR; two indicators): QSR indicators are based on routine data (up to one-year follow-up) for inpatient stays of AOK-insured patients [46]. AOK is the biggest sickness fund in Germany with around 20.6 million insured persons and more than 36% of the statutory health insurance market in 2018 [48]. The WIdO, the scientific institute of the AOK, uses AOK routine patient data to calculate hospital-level O/E-ratios. It performs annual risk-adjustment by means of logistic regression using Huber-White Sandwich Estimators that include patient-specific risk-factors such as age, gender and co-morbidities (for variables and regression weights see methodological S1 Appendix or risk statistics of the institute) [49]. Indicators are available from 2004 to 2014 (see Table 1) and non-personal, hospital-level data are provided to research groups for specific research projects upon application.

Several research and quality assurance institutions, e.g. IQTIG, set a minimum number of cases per hospital when evaluating and publishing results in order to minimise the influence of chance and assure statistical reliability [18, 46, 50, 51]. Outcome indicators for small case volumes and rare events per hospital would otherwise be biased by statistical confounding factors [16, 52–56]. Similarly, this study introduced a minimum case volume criterion. Since case volumes vary greatly across treatment areas, indicator-specific minimum volumes were defined rather than using a general case volume limit for all indicators (see S4 Appendix). Based on Calderwood *et al*., [18] hospitals for which $case\;volume\;per\;year<\frac{1}{average\;observed\;rate\;for\;indicator}$ in at least one year were excluded from the datasets. For these hospitals, average case volumes remain small across all years and most meet the exclusion criteria for many if not all years. Due to this requirement, the proportion of excluded hospitals varied between about 11% for community-acquired pneumonia (PNEU), decubitus ulcer (DECU), cholecystectomy (CHOLEC) and hip fracture (HIPFR) to about 40% for STROKE and hip replacement (HIPREPDI) (see S3 Appendix).

After applying the minimum case volume to each treatment area, we had an unbalanced dataset with missing data, and, thus, developed a second balanced dataset for which hospitals without complete documentation were excluded. The analyses were performed for both datasets and representativeness of the unbalanced dataset based on the full population was confirmed (see S3 Appendix).

### Methodological approach

The stability over time of patient-relevant hospital quality quintiles in Germany was explicitly evaluated in this longitudinal, observational survey. Whether a hospital produces better-than-average quality or is among the top 20% nationally is more useful for decision-making than individual hospital rankings, due to relatively large confidence intervals and small differences across single ranking positions (e.g., if a hospital is first or third). The use of quintiles as quality clusters is based on the AOK Hospital Navigator, projects from the USA and several other publications [8, 16, 57]. In order to form quality quintiles per indicator, hospitals in the balanced and unbalanced datasets were ranked in ascending order according to their O/E-ratio per year, and divided into performance quintiles with the same number of hospitals per quintile. Furthermore, the hospitals were sorted by average case volume across all years together and divided into three same-sized volume categories with (1) lowest, (2) mid-range and (3) highest case volume per indicator (for volume ranges per category see S4 Appendix).

A logistic regression was performed using Generalized Estimating Equations (GEE), which is particularly applicable for time-correlated data series [58]. The logistic regression using GEEs enables the measurement of the relative fluctuation of hospital performance with repeated measures over time, thus capturing relative stability of quality indicators [18, 24, 59, 60]. Compared to the methodology of Roshanghalb et al., [27] which used funnel plots to determine stability of (few) hospitals with outstanding good/bad outcomes, these quintiles classified hospitals based on their performance and allowed for a broader definition of good/bad hospitals. Additionally, graphical descriptive analysis and Spearman’s rank correlation coefficients were used to validate the results of our primary method (GEE).

The GEE method is an extension of the Generalized Linear Models (GLM). Its application generates non-biased estimates for correlated variables [61–63].

For the graphical descriptive analysis, we decided on a methodology that has already been used in similar studies [16, 17, 37]. It is an extension of the comparison of quality quintiles, as used, e.g., by Pross *et al*. [8] to highlight quality variation, with our analysis adding a time-dimension. Furthermore, Spearman’s rank correlation was used to determine the strength of correlation between last year’s and this year’s ranking of a hospital. While the graphical descriptive analysis delivered a more intuitive graphical impression of absolute stability, logistic regression using GEE provided information on relative stability of the hospitals.

### Data analysis

#### Graphical descriptive analysis

The graphical analysis of the indicators presents the average current performance (t) of the performance quintiles based on hospital rankings across different years. For each year we created three different quintile sets based on current (t) and past hospital quality rankings (t-1 and t-2). We took the hospitals within these quintile sets across each year (t-2, t-1, t) and calculated current average performance. The average case volume per hospital across all reported years was used for weighting. In this way, it was possible to estimate the usefulness of past quality quintiles to predict current performance. To enable intuitive interpretation of average hospital performance, the risk-adjusted rate (RAR) was calculated: $RAR=\frac{O}{E}\;ratio*overall\;mortality\;rate(across\;all\;hospitals\;in\;Germany)$.

#### Spearman`s rank correlation

A corrected formula, which takes into account the ties between ranking lists, was used for the calculating the Spearman’s rank correlation [64]. If positions within a variable were identical, average ranking positions were formed:

$$r_{s}=\frac{\sum_{i=1}^{n}{rank(x_{i})}^{2}+\sum_{i=1}^{n}{{rank(y}_{i})}^{2}-\sum_{i=1}^{n}{d_{i}}^{2}}{\sqrt[{2}]{\left(\sum_{i=1}^{n}{rank(x_{i})}^{2})*(\sum_{i=1}^{n}{rank(y_{i})}^{2}\right)}}$$

$$,with\;rank(x_{i})=rank\;of\;hospital\;i\;in\;the\;first\;year\;(x)$$

$$rank(y_{i})=rank\;of\;hospital\;i\;in\;the\;following\;year\;(y)$$

$$d=rank(x_{i})-rank(y_{i})$$

#### Generalized Estimating Equations (GEE)

To model the outcome of being in the best quintile this year (t) based on best quintile status last year (t-1), GEE logistic regression was used for all hospitals combined and by volume category for each indicator.

A logit function was selected as the link function and binomial distribution was chosen as the distribution of dependent variables, based on recommendations from Ballinger: [65]

$$logit(\mathrm{Pr}\left[Y_{i,t}=1\right])=\frac{Pr[Y_{i,t}=1]}{1+Pr[Y_{i,t}=1]}=\beta_{0}+\beta_{1}*{q1}_{i,t-1}+\beta_{2}*{q2}_{i,t-1}+\beta_{3}*{q3}_{i,t-1}+\beta_{4}*{q4}_{i,t-1}$$

$$,with\;Y_{i,t}=hospital\;i\;in\;quintile\;1\;in\;year\;t\;(binary),$$

$${q1}_{i,t-1}=hospital\;i\;in\;quintile\;1\;in\;year\;t-1\;(binary),$$

$${q2}_{i,t-1}=hospital\;i\;in\;quintile\;2\;in\;year\;t-1\;(binary),$$

$${q3}_{i,t-1}=hospital\;i\;in\;quintile\;3\;in\;year\;t-1\;(binary),$$

$${q4}_{i,t-1}=hospital\;i\;in\;quintile\;4\;in\;year\;t-1\;(binary)$$

Since the dataset comprised longitudinal data with equidistant measurement times, for which higher correlations were expected in closer years within a subject, a first-order autoregressive model was used for the working correlation matrix. A sandwich-estimator was used as it is largely robust against misspecification of the working correlation matrix [61, 63]. The standard Wald test was performed to test for significance of the coefficients [66].

Due to the MCAR-requirement, we primarily ran the analysis on the balanced dataset and based our discussion on its results. Furthermore, we used the unbalanced and imputed balanced to test sensitivity and robustness of the results and to get an impression of whether the findings could be extended to all hospitals.

All calculations were performed with IBM SPSS version 25 (64-bit) and R version 3.6.1 (64-bit). A more in-depth discussion of the methods employed and the variables used for risk-adjustment can be found in a methodological appendix (see S1 Appendix).

### Sensitivity analysis

A systematic imputation of extreme values into the data gaps of the unbalanced dataset was simulated to ensure robustness of results. For this purpose, previous year’s values from the hospitals as simulation of greatest possible stability (best case scenario), normally distributed random values (worst case scenario), and maximum values (scenario with systematic restraint of bad values by hospitals) were used to replace missing values.

## Results

### Descriptive analysis

The descriptive data in S2 Appendix found that indicators are heterogeneous, with relevant differences in event rate (0.39%/DECU to 14.85%/AMI), outcome variation (0.44/PNEU to 1.32/HIPREPDI and average case volume per hospital (68.8/AMI to 11,668.2/DECU). The differences between balanced and unbalanced datasets were small with regards to indicator and hospital characteristics (see S2, S3 Appendices). The number of data points considered varied between indicators (1,992/HIPREPDI to 10,492/AMI data points) due to different survey periods and number of hospitals (664/HIPREPDI (balanced) to 1.451/DECU (unbalanced)). Finally, the indicators HIPREPDI and HIPREPRE had a relatively high proportion (over 20% of observations) of zero values, meaning that no observed events in the survey period of the hospital occurred.

### Graphical descriptive analysis

**Fig 1:**
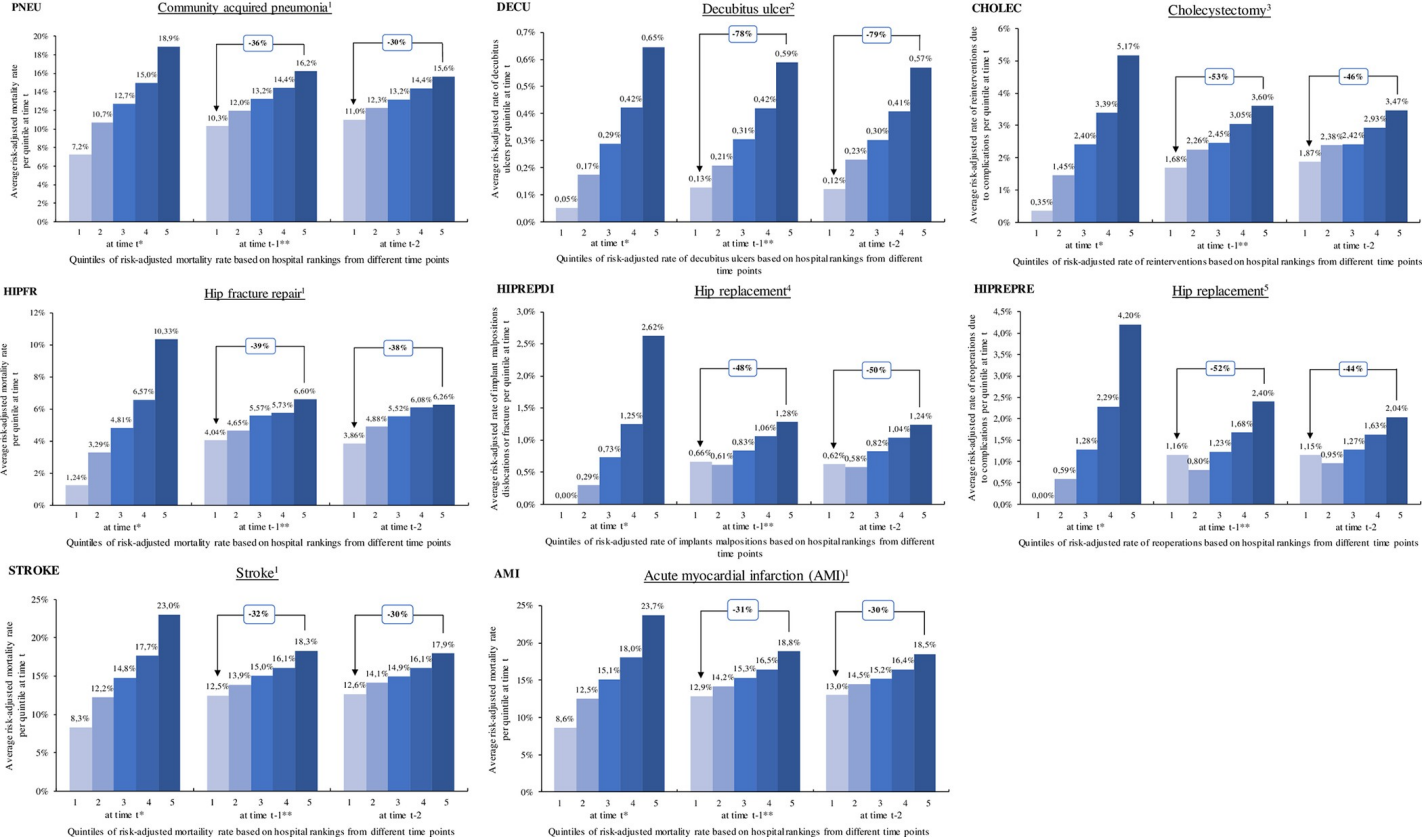
Average hospital performance per quality quintile (RAR) in t for quintiles based on hospital rankings in t, t-1 and t-2 (all hospitals; balanced dataset). Notes: Average performance as risk-adjusted rate (RAR) of all hospitals in a quintile at time t for quality quintiles assembled on the basis of the hospital rankings at time t (left block), t-1 (middle block) and t-2 (right block). Quintiles within a block are sorted in ascending order from left best quality (light blue) to right worst quality (dark blue). 1 SMR 2 rate of decubitus ulcer (acquired stationary) 3 rate of reinterventions (complications) 4 rate of implant malpositions, dislocations or fractures 5 rate of reoperations (complications). * The first two available years have been excluded from the calculation to ensure full comparability of the calculations. ** The first available year has been excluded from the calculation to ensure full comparability of the calculations.

Fig 1 shows the average quality of hospitals in year t per quality quintile with hospitals sorted into quintiles based on quality in year t (left bar chart of each indicator specific- graphic). The middle and the right chart for each indicator show the current quality of hospitals that were grouped in quintiles based on historical hospital quality in t-1 (middle) and t-2 (right), respectively.

The stability of quality decreased with increasing time interval. For example, the difference of risk-adjusted rate for HIPREPRE and CHOL from quintile 5 in comparison to quintile 1 was 52% and 53% based on one-year old as well as 44% and 46% for quintiles based on two-year old hospital rankings. The decrease of the difference over time was small, however. No difference was observed for the two indicators HIPREPDI and DECU.

Overall, patients selecting a hospital today (t) would experience on average a reduction in the rate of adverse events between 30% (PNEU and AMI) and 79% (DECU), when choosing a hospital from quintile 1 instead of quintile 5 based on two-year old hospital performance quintiles. If a top hospital was selected based on previous year’s (t-1) indicator results, the reduction in adverse event rates was similarly more than 31% for all indicators. As quintile selection changed every year, current risk-adjusted rates at time t for the best quintile of year t were better than current rate for the best quintile of year t-1 (e.g., 1.24% vs. 4.04% for HIPFR or 8.6% vs. 12.9% for AMI).

### Spearman’s rank correlation

A positive monotonic correlation between this year’s and last year’s hospitals ranking, significant at the 1% level (p<0.01), was found for all indicators. Therefore, a better ranking last year indicated a better ranking this year. The calculated coefficients ranged from 0.234 for HIPREPDI to 0.798 for DECU. They were relatively low–between 0.234 and 0.329 –for surgical indicators and the emergency treatment areas STROKE and AMI.

### Generalized Estimating Equations (GEE)

**Fig 2:**
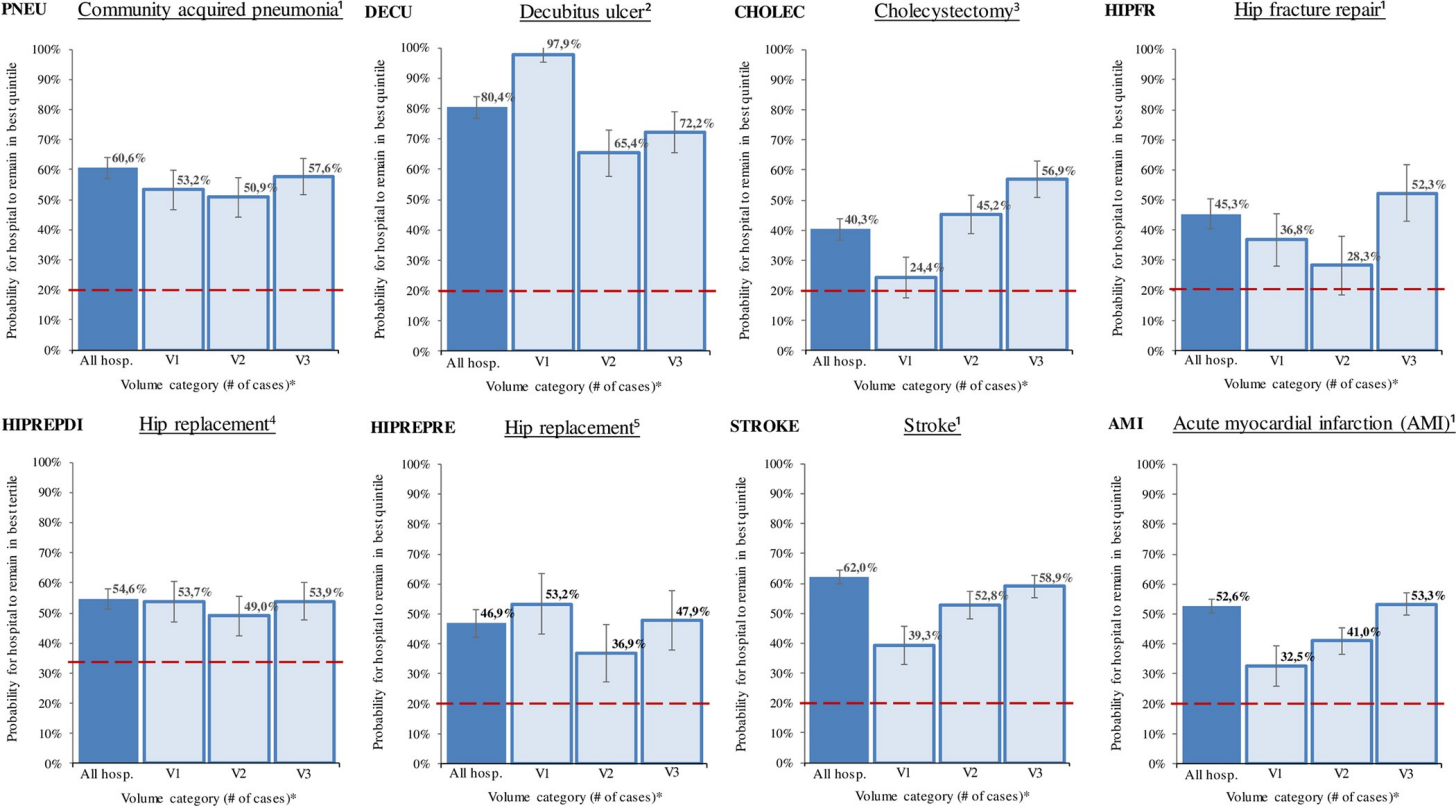
Probability of best quintile ranking this year (t) for a hospital with a best quintile ranking last year (t-1) (balanced dataset; all hospitals and per volume category). Notes: Probabilities of remaining in the best quintile/tertile next year for hospitals with a best quintile/tertile ranking this year presented with 95% confidence intervals for each indicator and different volume categories. The volume category corresponds to the third of the hospitals with the fewest cases (V1), mid-range case volume (V2) and highest case volume (V3). All hospitals were assigned to exactly one category based on their average number of cases across all years. (see Table (S4 Appendix). A logistic regression model using generalized estimating equations (GEE) was conducted to determine the probabilities. The dashed red line represents the state of chance. It is equal to 20% when using quintiles and 33% when using tertiles (HIPREPDI). 1 SMR 2 ratio of decubitus ulcer (acquired stationary) 3 ratio of reinterventions (complications) 4 ratio of implant malpositions, dislocations or fractures 5 ratio of reoperations (complications).

Fig 2 shows the probabilities calculated with logistic regression using GEEs that a hospital classified in the best quintile (quintile 1) in one year (t-1) would sort into the best quality quintile the following year (t) per volume category. For indicator HIPREPDI, a logistic regression model could not be adapted to quintiles for every volume category as more than 20% of hospitals (in particular, small clinics) had a quality indicator value of zero. Therefore, tertiles were used for this indicator.

The probability of remaining in the best quality quintile (or tertile) in the future for each quality indicator across all hospitals differed significantly from chance (which would be 20% for quantiles and 33% for terciles, see dashed lines in Fig 2), since 95% confidence intervals (CI) did not overlap with these chance values, ranging from 46.9% (CI: 42.4–51.6%) for HIPREPRE to 80.4% (CI: 76.4–83.8%) for DECU. The determined stability was lowest for the surgical indicators CHOLEC, HIPFR, HIPREPDI (tertiles) and HIPREPRE.

A significant stability was also found for all case volume categories and indicators except for the lowest volume group of the indicators CHOLEC (24.4% (CI: 16.4–34.5%)) and the mid volume group HIPFR (28.3% (CI: 19.6-39%)). The indicators CHOLEC, STROKE and AMI showed a continuous increase in stability over time with increasing case volume from 24.4/39.3/32.5% for hospitals with smallest case volumes to 56.9/58.9/53.3% for hospitals with largest case volumes. Thus, hospitals with a higher case volume are more likely to remain in the top quintile. The remaining indicators PNEU, DECU, HIPFR, HIPREPDI and HIPREPRE yielded the lowest stability over time in the medium volume category.

### Sensitivity analysis

When comparing the balanced and unbalanced datasets in Table 2, there were only small differences in the estimated probabilities and confidence intervals. The maximum difference was two percentage points for the mortality indicators for the treatment areas STROKE and AMI.

**Table 2:** Comparison of the balanced, unbalanced and simulated extreme value scenarios (imputation of missing values; sensitivity analysis).

Indicator abbreviation	Orig. ID	Indicator description (short)	Examined quality cluster	Probability of remaining in same quintile (95% confidence interval in brackets)				
				Balanced	Unbalanced	Imputation (simulation of missing ratios)		
						Identical to the previous year^1^	Normal distribution^2^	Maximum ratios^3^
PNEU	50778	Community acquired pneumonia; SMR	Quintile 1	0.606* (0.57–0.64)	0.595* (0.561–0.628)	0.685* (0.654–0.714)	0.589* (0.56–0.618)	0.600* (0.571–0.629)
			Quintile 5	0.598* (0.565–0.631)	0.582* (0.551–0.614)	0.636* (0.607–0.664)	0.564* (0.535–0.594)	0.592* (0.563–0.62)
DECU	52009	Decubitus ulcer; ratio of ulcers acquired in hospital	Quintile 1	0.804* (0.764–0.838)	0.809* (0.769–0.843)	0.860* (0.83–0.886)	0.721* (0.685–0.754)	0.709* (0.673–0.743)
			Quintile 5	0.701* (0.657–0.741)	0.685* (0.642–0.725)	0.733* (0.696–0.767)	0.650* (0.611–0.687)	0.527* (0.488–0.565)
CHOLEC	50791	Cholecystecomy; ratio or reinterventions	Quintile 1	0.403* (0.354–0.455)	0.391* (0.342–0.442)	0.440* (0.392–0.49)	0.391* (0.344–0.441)	0.390* (0.343–0.44)
			Quintile 5	0.393* (0.345–0.443)	0.398* (0.35–0.447)	0.429* (0.382–0.477)	0.381* (0.335–0.429)	0.384* (0.339–0.432)
HIPFR	51168	Hip fracture repair; SMR	Quintile 1	0.453* (0.404–0.503)	0.453* (0.405–0.502)	0.478* (0.431–0.526)	0.434* (0.387–0.483)	0.450* (0.403–0.498)
			Quintile 5	0.339* (0.294–0.388)	0.338* (0.294–0.384)	0.367* (0.322–0.414)	0.334* (0.291–0.379)	0.335* (0.292–0.382)
HIPREPDI	50919	Hip replacement; ratio of implant dislocations	Tertile 1	0.546* (0.5–0.591)	0.543* (0.497–0.588)	0.570* (0.527–0.613)	0.519* (0.474–0.563)	0.521* (0.475–0.565)
			Tertile 3	0.535* (0.489–0.58)	0.531* (0.486–0.575)	0.588* (0.545–0.631)	0.509* (0.464–0.553)	0.557* (0.512–0.601)
HIPREPRE	50944	Hip replacement; ratio of reoperations	Quintile 1	0.469* (0.424–0.516)	0.464* (0.419–0.511)	0.498* (0.454–0.542)	0.464* (0.419–0.509)	0.457* (0.412–0.502)
			Quintile 5	0.435* (0.385–0.486)	0.427* (0.378–0.478)	0.454* (0.406–0.502)	0.423* (0.375–0.472)	0.412* (0.366–0.461)
STROKE	2002	Stroke; SMR	Quintile 1	0.620* (0.591–0.643)	0.599* (0.576–0.621)	0.721* (0.699–0.743)	0.586* (0.565–0.606)	0.600* (0.579–0.621)
			Quintile 5	0.588* (0.563–0.612)	0.592* (0.569–0.615)	0.711* (0.687–0.734)	0.537* (0.515–0.558)	0.851* (0.832–0.869)
AMI	2001	AMI; SMR	Quintile 1	0.526* (0.504–0.548)	0.505* (0.484–0.527)	0.663* (0.638–0.687)	0.473* (0.453–0.493)	0.519* (0.498–0.54)
			Quintile 5	0.564* (0.54–0.588)	0.561* (0.538–0.584)	0.675* (0.651–0.698)	0.534* (0.514–0.555)	0.827* (0.808–0.845)

Notes:

Probabilities of remaining in the best/worst quintile/tertile next year for hospitals with a best/worst quintile/tertile ranking this year presented with 95% confidence intervals in brackets for each indicator and different datasets. A logistic regression model using generalized estimating equations (GEE) was conducted to determine the probabilities.

* The probabilities have 95% confidence intervals excluding the null value of 0.2 for quintiles and 0.33 for tertiles (only HIPREPDI)–the probability of remaining in the quintile/tertile by chance.

^1^ Best case scenario: Imputation of previous year’s values from the hospitals

^2^ Worst case scenario: Imputation of normally distributed random values

^3^ Scenario of the intentional withholding of bad results by hospitals: Imputation of maximum values of the specific indicator during the observation time

The results for indicator stability over time of hospitals with the worst outcome quality (quintile 5) were mostly consistent with indicator stability over time for hospitals with the best outcome quality (quintile 1).

When simulating three scenarios by imputing missing ratios, probabilities remained at the same level. The simulation of hospitals intentionally withholding bad results just decreased the calculated probabilities of remaining in the best quintile by up to two percentage points for all indicators, except DECU.

Finally, the simulation of large random influence in missing values resulted in a lower stability and decreased probabilities of around two to five percentage points for most indicators. DECU, on the other hand, saw a decrease of around eight percentage points, though stability over time of this indicator nevertheless remained at a high level.

## Discussion

This study sought to determine whether eight hospital quality indicators in Germany are stable over time and as such have predictive informational value for the current or future quality of a hospital. Data were obtained from the G-BA and QSR hospital quality report cards between 2004 to 2017 with a survey period of up to ten years per indicator. For an intuitive interpretation of results, a logistic regression using GEE was used, and results were validated by Spearman’s rank correlation coefficient and a time-dependent, graphic quintile representation of risk-adjusted rates (RAR). This graphical analysis has been applied previously in similar analyses [16, 17, 37] and helps to illustrate the results. The analysis expands the methodological framework to evaluate stability overtime with a GEE application, which can utilize hospital-level, aggregated, non-reliability adjusted quality indicators. Importantly, this is the type of data which is used for public reporting and is often available for research due to data privacy and regulatory concerns.

Overall, the results demonstrated some significant stability over time for a wide range of quality indicators as the calculated values differ significantly from chance. However, there were important differences across indicators. For example, the risk-adjusted decubitus ratio (DECU) demonstrated a relatively high amount of stability, whereas the mortality ratios of surgical interventions have weaker stability. Results from the primary method GEE were consistent with results obtained from other methods employed as robustness checks.

The level of stability over time found in this study partly contrasts with the results of a first study using GEE. Calderwood *et al*. [18] found a probability of remaining in quartile 4 for postoperative infections of 39% (hip replacement) and 44% (CABG) across all hospitals. In this context, the researchers concluded probabilities of up to 59% as too low to qualify as sufficiently stable over time. They further concluded that the considered indicators were not suitable as a basis for value-based purchasing. However, their study made use of quartiles (random probability is 25%) instead of quintiles (random probability is 20%) and employed a reliability adjustment, which shifted values of hospitals with small case volumes and big uncertainty to the mean [18]. In fact, both conditions would have led to better results compared to the our study if the underlying, non-reliability-adjusted quality indicator data had been applied [17, 67]. As it is, this study indicated higher stability over time for these indicators: The probabilities for the non-surgical indicators DECU, PNEU and STROKE are substantially higher than the maximum probability of the study conducted by Calderwood *et al*., while the results for surgical indicators were more similar [18].

The differences in predictive probabilities across indicators can partly be explained by their varying discrimination power [33, 54, 55, 68]. Specifically, IQTIG and aQua estimate the indicators DECU, PNEU and CHOLEC as good and HIPFR, HIPREPDI and HIPREPRE as moderate in terms of their discrimination power [69–71]. The two institutes furthermore developed a minimum volume per indicator to achieve good discrimination power. For DECU and PNEU at least 80% of hospitals including in the study met their respective requirement. In contrast, less than 20% of the study hospitals achieved the minimum case volume thresholds of aQua and IQTIG for surgical interventions (CHOLEC, HIPFR, HIPREPDI and HIPREPRE) [69, 70]. The weaker stability of the surgical quality indicators may therefore largely be attributed to the larger random influence associated with lower surgical volumes. The sample prevalence problem, which describes a strong negative relationship between the impact of chance and hospital case volume/event rate of an indicator, has been confirmed in several German and international studies [54, 72, 73]. Due to a lack of specialisation and the large number of hospitals in Germany, hospitals have relatively low average annual case volumes–between 100 and 250 –for many surgical interventions that also have low event frequencies (between 1% and 5%). Meanwhile, the more stable indicators are shown to either have higher average case volumes (e.g. 11,668 for DECU) or higher event rates (e.g. 14.85% for AMI) and are thus less susceptible to the impact of chance (see S2 Appendix).

Previous studies have highlighted the relationship between the high impact of chance and low stability [52, 60]. Our findings are mostly in line with Birkmeyer *et al*., [16] who derived small case volumes as the cause for low predictive power and the limited ability to represent true quality using the mortality rate of esophageal resections. Small case volumes in combination with low event rates remain a significant limitation in this study. Although surgical case volumes for many hospitals were below minimum case volume for adequate discrimination, the calculated stability over time still deviates significantly from chance, similar to findings from Krell *et al*. [60].

Meanwhile, regarding the sample prevalence problem, the positive relationship found between case volume and stability can be partly explained by reduced statistical chance because of higher case volume. In particular, the indicators CHOLEC, STROKE and AMI revealed a continuous and significant improvement in stability with increasing average annual case volume among all three methods used. For HIPFR, HIPREPDI and HIPREPRE, however, there is no such clear, continuous relationship. In some cases, the low-volume category even shows better stability over time than the high-volume category in the logistic regression. However, on closer examination of the data, this seemingly counterintuitive result is not contradictory as the datasets of the mentioned indicators have a high proportion of zero values. These results are achieved when no adverse event has been observed. For the low-event, surgical outcome indicators, such results are mostly achieved by facilities with low case volume, not due to high quality of care, but due to statistical chance. In fact, for many surgical interventions low case numbers are on average associated with poorer treatment quality [74–76].

### Policy implications

Several policy implications can be drawn from the results of this study, which are relevant not only in Germany, but also for other healthcare systems investing in outcome quality assessment and, especially, transparency like the UK and the USA. These fall within three categories: public reporting of quality of hospital care; minimum surgical volume-quantity regulation and centralisation of hospital services; and outcome-based contracting.

#### Public reporting

As a criterion in the decision-making process related to care utilisation (but also delivery and organisation), access to information on outcome quality could lead to improved hospital choice. Patients and referring doctors as well as health insurances can use the information of this study to choose, or inform choices around, higher quality hospitals and avoid lower quality hospitals. Moreover, the stability over time of quality indicators demonstrated by this study may help to encourage clinicians to support current and future quality transparency initiatives more actively, contribute to their improvement, and, most importantly, discuss the results with their patients as part of shared decision-making.

In order to achieve this goal, the results have shown that the introduction of an indicator-specific minimum sample size for public reporting is essential. Even after introducing a minimum case volume for hospitals in the data set, the examination of specific volume categories has shown that the stability of the included hospitals in quintile 1 over time can be close or equal to chance for hospitals with small case volumes and significantly increases with average case volume of the examined hospitals. The volume cutoffs currently are often based on parameters as event rate, confidence intervals or fixed cross-indicator minimum case volumes [18, 46, 50, 51]. To ensure patient benefit, it is recommended that stability (e.g. deviation from chance) be included as a minimum requirement in the catalog of criteria for the publication of quality indicator results.

#### Minimum surgical volume regulation and centralisation of hospital services

The positive relationship between outcomes and case volumes are already the basis for minimum surgical volume regulation in many countries. Hospitals with very low case volumes in a respective treatment area due to a presumed lack of clinical experience and expertise and lower quality than in more specialized centers [74–77]. Next to this positive relationship between outcomes and volumes, this paper also highlights the positive relationship between stability of hospital quality indicators and case volumes. Higher case volumes improve the signal to noise ratio for indicators with high statistical uncertainty and thus further enhance the policy dividends of such regulation by boosting the information and decision value in public reporting.

#### Outcome-based contracting

When considering the expansion of outcome-based contracting in healthcare, stability over time is a crucial factor in determining the suitability of quality indicators. It must be ensured that the derived actions for hospitals based on historical indicator results are on average accurately reflecting current quality of care. In particular, decisions with serious consequences, such as a treatment ban or a reduction of reimbursements if quality is insufficient, can hardly be justified on the basis of one or two-year-old data if the measured quality fluctuates sharply every year.

From the analyzed quality indicators, indicator such as DECU and PNEU tend to be better suited as a basis for decision-making for outcome-based payment and contracting as they show a stability in their quintile ranking for the majority hospitals. Our findings support those of other studies that found that justification of such drastic interventions to single outcome indicators without very high stability over time is not recommended [18, 34]. Thus, it is absolutely necessary to systematically evaluate the usefulness of every indicator with regards to sufficient stability over time, e.g. for example with stability being substantially different than chance, for outcome-based payment schemes.

### Strengths and limitations

By including all valid cases from at least 664 to 1,451 hospitals per indicator in Germany, this study exceeds all the analyzed predecessor studies in terms of indicator and time coverage. For two indicators, the time span covered ten years, which is long compared to previous research. Another strength is the accessible presentation of the results via quintiles (and tertiles) and the respective stability over time of a hospital’s position. Therefore, it is also possible for non-experts, such as informed patients or interested doctors, to derive their own conclusions and considerations. Lastly, results are highly consistent across methods used and the GEE method’s benefits for analyzing stability over time of outcome indicators are demonstrated.

The susceptibility to manipulation for publicly, self-reported data, as comprises the German Mandatory National Quality Monitoring, is well known [78]. Hospitals have a natural interest in maintaining a good reputation. The IQTIG performs complex data validation to prevent this form of manipulation [72, 79]. Furthermore, the AOK QSR is based on administrative data, so the quality indicators are less susceptible to manipulation.

Missing values in the dataset represent a significantly greater limitation for reliable results and transferring the results to all hospitals. Some hospital results were not published in the case of low case numbers for data privacy reasons. Furthermore, hospitals merged and closed in the consolidating German hospital market in the intervening time, and documentation errors also occurred. In general, the hospitals are obliged to report data as part of mandatory quality monitoring. Since it was not possible to verify the MCAR requirement of the GEE, we had to exclude hospitals without a full data set across all years and have run the GEE on the balanced data set. In this context, we additionally performed several sensitivity analyses using the complete unbalanced dataset and imputed balanced datasets to ensure that the stability over time was not overestimated due to systematic dropout of hospitals and thus missing data in the dataset and to rule out misleading interpretations. In particular, it was shown that the possibly systematic withholding of poor results by hospitals would not lead to a noticeable deterioration in the stability estimate for the best hospitals. For all indicators, despite simulated changes in the dataset, adequate robustness of the results was demonstrated. In single years, there were no results in the underlying dataset of the G-BA from Rhineland-Palatinate and several parts of North Rhine-Westphalia available due to privacy concerns. Those hospitals were excluded. There is no indication that there are serious peculiarities in the hospital landscape or population that lead to strong deviations of these regions from the rest of Germany.

Lastly and importantly, as we don’t have access to information about hospitals’ quality improvement strategies, we cannot adjust for clinical improvement initiatives at the hospital level. Some hospitals might have purposely improved their quality, e.g. for indicators such as decubitus, and thus switched quality quintiles, which would have been an intentional reduction in stability. Since this study only uses patient risk-adjusted, hospital level, aggregated quality indicators, we cannot differentiate between patient and hospital level affects, but since the data is risk-adjusted, the influence of patient risk factors is limited. Furthermore general regression to the mean, which was shown to be present in other studies for comparable indicators [80], has likely also occurred in our study and therefore decreased stability over time.

## Conclusion

Hospital quality stability over time with reliable information value is one of the essential requirements for the practical use of quality indicators in healthcare, esp. with regards to public reporting to support hospital choice. This study found that all the evaluated quality indicators have some stability over time. However, the strength varies greatly between the individual indicators and depends, for example, on the average case number per hospital. The different results demonstrate the need for an indicator specific stability assessment, with potentially using the requirement of stability over time having to be substantially different from chance as a requirement in designing minimum volume thresholds.

With an adapted GEE application, this study expands the methodological framework to examine quality indicator stability over time with a transferable, easy-to-use and relevant applied method. This applied method also takes up directly the data used for public reporting, thus the method and stability over time results benefit patients, admitting physicians and policy makers using this data for current hospital choice decisions. Future research should examine in more detail potential indicator stability requirements depending on different policy interventions, examine potential changes in stability over time and test the method with hospital level data from other countries.

## Supporting information

S1 Appendix Methods and models. (PDF)

S2 Appendix Statistical characteristics of the variables outcome and volume in the (unbalanced and balanced) hospital quality indicator data set. (PDF)

S3 Appendix Characteristics of the included hospitals per indicator and data set (balanced and unbalanced). (PDF)

S4 Appendix Indicator-specific minimum case volume and case volume categories (Cat.) per quality indicator. (PDF)
