# Supplementary material for: Stability of hospital quality indicators over time: A multi-year observational study of German hospital data
Source: PLoS One. 2023 Nov 7;18(11):e0293723. doi: 10.1371/journal.pone.0293723 (PMC10629650; doi:10.1371/journal.pone.0293723)
Supplement: S1 Appendix — (PDF) [file pone.0293723.s001.pdf]

APPENDIX 1: Methods and models

Variables for risk-adjustment by treatment area – risk-adjustments undertaken by the institutes that have provided the data for secondary analysis

PNEU: age, gender, admission from inpatient care facility, admission from another hospital or from inpatient rehabilitation facility, chronic bedridden status, invasive mechanical ventilation on admission, disorientation upon admission, spontaneous respiratory rate, blood pressure systolic on admission, difference between systolic and diastolic blood pressure on admission

DECU: age, gender, diabetes mellitus, hours of ventilation, paraparesis and paraplegia, tetraparesis and tetraplegia

CHOLEC: age, gender, ASA classification, open surgery, gallstones, empyema, gallbladder perforation, contracted gallbladder

HIPFR: age, gender, ASA classification, CDC wound classification, localisation of fracture, Garden classification

HIPREPDI: gender, ASA classification, CDC wound classification, pre-operation on the hip joint

HIPREPRE: age, gender, ASA classification, CDC wound classification, pre-operation on the hip joint

STROKE: age, gender, type of stroke, atrial fibrillation, atherosclerosis, heart failure, dilated cardiomyopathy, atrioventricular block, valvular heart disease, chronic renal failure, severe kidney disease, metastasis, ventricular tachycardia

AMI: age, gender, type of acute myocardial infarction, stroke, atherosclerosis, heart failure, dilated cardiomyopathy, cardiogenic shock, atrioventricular block, apoplexy, severe kidney disease, metastasis, ventricular tachycardia, ventricular fibrillation

Spearman rank correlation

In preparation for the analysis, all hospitals were ranked for each year according to their O/E-ratio in ascending order from 1 (lowest O/E ratio) to n (highest O/E ratio).

A corrected formula[1] was used for the calculation, which takes into account ties between hospital. If positions within a variable are identical, average ranking places are formed.

$$r_s = \frac{\sum_{i=1}^{n} rank(x_i)^2 + \sum_{i=1}^{n} rank(y_i)^2 - \sum_{i=1}^{n} {d_i}^2}{\sqrt[2]{(\sum_{i=1}^{n} rank(x_i)^2) * (\sum_{i=1}^{n} rank(y_i)^2)}}$$

$, with\ rank(x_i) = rank\ of\ hospital\ i\ in\ the\ first\ year\ (x)$

$rank(y_i) = rank\ of\ hospital\ i\ in\ the\ following\ year\ (y)$

$d = rank(x_i) - rank(y_i)$

Thereby, $\sum_{i=1}^{n} rank(x_i)^2$ and $\sum_{i=1}^{n} rank(x_i)^2$ are the sums of the squared rankings of hospital i in the first year (x) and the following year (y). They are calculated with the following formula:

$$\sum_{i=1}^{n} rank(x_i)^2 = \sum_{i=1}^{n} rank(y_i)^2 = \frac{n^3 - n}{12} - \sum_{k=1}^{t_{i,k}} T_k$$

, where $T_k = \frac{1}{12} * \sum_{i=1}^{k} (t_k{}^3 - t_k)$

In this formula, t_k is the number of ties on a rank. There are several ties in one variable. These are considered sequentially according to index k.

The test for significance with the null hypothesis $r_s = 0$ was conducted using the t-distributed variable $t = r_s * \sqrt{\frac{n-2}{1-r_s{}^2}}$ (critical t-value at the 1%-level; $df = n - 2$). If n is high, as in the examined datasets, this returns exact results.[2]

Logistic regression using GEE

Based on the hospital ranking, quality quintiles were formed with the same number of hospitals in each quintile. These quintiles were initially used to create the binary variable $Y_{i,t+1}$. If a hospital i in year t + 1 was in quintile 1, then $Y_{i,t+1} = 1$. Otherwise, $Y_{i,t+1} = 0$. The same procedure was used for the binary predictor variables. If a hospital i in year t was accordingly in quintile 1, then $q1_i = 1$ and, thus, $q2_{i,t} = q3_{i,t} = q4_{i,t} = q5_{i,t} = 0$ and so on. Only binary variables were used in the model.

The logistic regression using Generalized Estimating Equations (GEE) used in this work was developed by Liang and Zeger.[3, 4] The formula to calculate the probability of best quintile ranking this year (t) for a hospital with a best quintile ranking last year (t-1) for the indicators PNEU, DECU, CHOLEC, HIPFR, HIPREPRE, STROKE and AMI can be expressed as follows:

$$logit(\Pr[Y_{i,t} = 1]) = \frac{\Pr[Y_{i,t} = 1]}{1 + \Pr[Y_{i,t} = 1]}$$
$$= \beta_0 + \beta_1 * q1_{i,t-1} + \beta_2 * q2_{i,t-1} + \beta_3 * q3_{i,t-1} + \beta_4 * q4_{i,t-1}$$

$, with\ Y_{i,t} = hospital\ i\ in\ quintile\ 1\ in\ year\ t\ (binary),$

$q1_{i,t-1} = hospital\ i\ in\ quintile\ 1\ in\ year\ t - 1\ (binary)$,

$q2_{i,t-1} = hospital\ i\ in\ quintile\ 2\ in\ year\ t - 1\ (binary),$

$q3_{i,t-1} = hospital\ i\ in\ quintile\ 3\ in\ year\ t - 1\ (binary),$

$q4_{i,t-1} = hospital\ i\ in\ quintile\ 4\ in\ year\ t - 1\ (binary)$

The variable $q5_{i,t}$ was excluded from the formula as a reference variable.

The logistic regression using GEE to calculate the probability of best tercile ranking this year (t) for a hospital with a best tertile ranking last year (t-1) for the indicator HIPREPDI can be expressed as follows:

$$logit(\Pr[Y_{i,t+1}=1]) = \frac{\Pr[Y_{i,t+1}=1]}{1+\Pr[Y_{i,t+1}=1]} = \beta_0 + \beta_1 * q1_{i,t} + \beta_2 * q2_{i,t}$$

$, with\ Y_{i,t} = hospital\ i\ in\ tertile\ 1\ in\ year\ t\ (binary),$

$q1_{i,t-1} = hospital\ i\ in\ tertile\ 1\ in\ year\ t-1\ (binary)\ ,$

$q2_{i,t-1} = hospital\ i\ in\ tertile\ 2\ in\ year\ t-1\ (binary),$

The variable $q3_{i,t}$ was excluded from the formula as a reference variable.

The linking algorithm and the distribution of the dependent variables have to be correctly specified to avoid biased results.[5, 6] In contrast, there is general robustness of the GEE models to misspecification of the working correlation matrix.[4]

In order to create a suitable model, specifications for (1) the link function, (2) the distribution of the dependent variable (necessary for the calculation of the variance as a function of the population mean) and (3) a working correlation matrix were specified.

A logit function was chosen as the link function (1). It is the standard link function for binary, dependent variables. As distribution of dependent variables (2) the binomial distribution was chosen. This was done in accordance with recommendations[6] for binary outcome variables. Since the data represents equidistant survey periods, where higher correlations are expected for events closer together in time within a subject, a first-order autoregressive model for the working correlation matrix (3) was chosen. In this case it is assumed that the correlation decreases exponentially with measurements further apart in time.[6, 7]

A major advantage of the GEE method is the correct determination of standard errors and confidence intervals for correlated data. For use with the GEE method, two types of standard errors are applicable. The model-based standard error, which is based on an estimated correlation matrix, as well as the Huber-White or Sandwich estimator, which is calculated based on variance of the cluster level and the effective number of subsets. In general, with availability of a sufficiently large data set, the sandwich estimator provides similarly or even more accurate standard errors than the model-based estimator.[8] Furthermore, the sandwich estimator is substantially more robust against faulty specifications of the working correlation matrix.[9–11] Therefore, a sandwich-estimator was used.

The 95% confidence intervals were calculated using the following formula:

$$Lower\ Bound\ CI(P(Y_i=1)) = \frac{e^{\left(x_i{}^T*\beta-1{,}96*\sqrt{x_i{}^T*\theta*x}\right)}}{1+e^{\left(x_i{}^T*\beta-1{,}96*\sqrt{x_i{}^T*\theta*x}\right)}}$$

$$Upper\ Bound\ CI(P(Y_i=1)) = \frac{e^{\left(x_i{}^T*\beta+1{,}96*\ \sqrt{x_i{}^T*\theta*x}\right)}}{1+e^{\left(x_i{}^T*\beta+1{,}96*\ \sqrt{x_i{}^T*\theta*x}\right)}}$$

, with θ = covariance matrix

The standard Wald test was performed to test for significance of the coefficients.[12]

All calculations were performed with IBM SPSS version 25 (64-bit) and R version 3.6.1 (64-bit).

REFERENCES

1 Myers JL, Well A. Research design and statistical analysis. Mahwah, N.J.: Lawrence Erlbaum Associates 2003.

2 Bryman A, Lewis-Beck MS, Liao TF. The Sage encyclopedia of social science research methods. Thousand Oaks: Sage 2004.

3 Zeger SL, Liang K-Y. Longitudinal Data Analysis for Discrete and Continuous Outcomes. *Biometrics* 1986;42(1):121–30.

4 Liang K-Y, Zeger SL. Longitudinal data analysis using generalized linear models. *Biometrika* 1986;73(1):13–22.

5 Pendergast JF, Gange SJ, Newton MA, et al. A Survey of Methods for Analyzing Clustered Binary Response Data. *International Statistical Review* 1996;64(1):89–118.

6 Ballinger GA. Using Generalized Estimating Equations for Longitudinal Data Analysis. *Organizational Research Methods* 2004;7(2):127–50.

7 Baltes-Götz B. Generalisierte lineare Modelle und GEE-Modelle in SPSS Statistics. Trier 2016.

8 Hanley JA, Negassa A, Edwardes MDd, et al. Statistical analysis of correlated data using generalized estimating equations: an orientation. *American journal of epidemiology* 2003;157(4):364–75.

9 Hilbe J, Hardin J. Generalized estimating equations for longitudinal panel analysis. In: Menard SW, ed. Handbook of longitudinal research: Design, measurement, and analysis, 1st edn. Amsterdam: Academic Press 2008:467–74.

10 Overall JE, Tonidandel S. Robustness of Generalized Estimating Equation (GEE) Tests of Significance against Misspecification of the Error Structure Model. *Biometrical Journal* 2004;46(2):203–13.

11 Pekár S, Brabec M. Generalized estimating equations: A pragmatic and flexible approach to the marginal GLM modelling of correlated data in the behavioural sciences. *Ethology* 2018;124(2):86–93.

12 Rotnitzky A, Jewell N. Hypothesis testing of regression parameters in semiparametric generalized linear models for cluster correlated data. *Biometrika* 1990;77(3):485–97.
