# Supplementary material for: Stability of hospital quality indicators over time: A multi-year observational study of German hospital data
Source: PLoS One. 2023 Nov 7;18(11):e0293723. doi: 10.1371/journal.pone.0293723 (PMC10629650; doi:10.1371/journal.pone.0293723)
Supplement: S2 Appendix — (PDF) [file pone.0293723.s002.pdf]

APPENDIX 2: *Statistical characteristics of the variables outcome and volume in the (unbalanced and balanced) hospital quality indicator data set*

Indicator	Indicator description (short)	Variable	Data set	n (data points)	$\bar{x}$	s	$x_{0.2}$	$x_{0.8}$	min	max	Rate[1] (%)
PNEU	Community acquired pneumonia; SMR; risk-adjusted	Outcome	Unbalanced	6,199	0.96	0.5	0.63	1.26	0	12.96	13.07
			Balanced	5,250	0.98	0.44	0.67	1.26	0	10.85	
		Volume	Unbalanced	6,199	183.5	133.1	77	273	8	1037	
			Balanced	5,250	197.7	131.7	93	286	8	1037	
DECU	Decubitus ulcer; ratio of ulcers acquired in hospital; risk-adjusted	Outcome	Unbalanced	4,158	0.85	0.68	0.28	1.33	0	11.03	0.39
			Balanced	3,456	0.88	0.63	0.37	1.34	0	6.05	
		Volume	Unbalanced	4,158	10,597.5	10,455	2,783	16,106	268	11,9110	
			Balanced	3,456	11,668.2	10,778	3,915	17,332	277	11,9110	
CHOLEC	Cholecystecomy; ratio or reinterventions; risk-adjusted	Outcome	Unbalanced	3,087	1.07	0.8	0.4	1.63	0	7.81	2.45
			Balanced	2,937	1.07	0.8	0.41	1.64	0	7.81	
		Volume	Unbalanced	3,087	171.4	81.8	102	233	42	619	
			Balanced	2,937	171.4	81.7	102	233	42	619	
HIPFR	Hip fracture repair; SMR; risk-adjusted	Outcome	Unbalanced	3,084	1.03	0.74	0.49	1.48	0	14.94	5.25
			Balanced	2,973	1.03	0.74	0.49	1.48	0	14.94	
		Volume	Unbalanced	3,084	105.3	55	59	147	21	411	
			Balanced	2,973	105.3	54.9	60	147	21	411	
HIPREDI	Hip replacement; ratio of implant dislocations; risk-adjusted	Outcome	Unbalanced	2,104	1.11	1.32	0	1.85	0	9.07	0.98
			Balanced	1,992	1.08	1.29	0	1.8	0	9.07	
		Volume	Unbalanced	2,104	247.2	197.6	133	318	102	2179	
			Balanced	1,992	247.6	197.8	134	321	102	2179	
HIPREPRE	Hip replacement; ratio of reoperations; risk-adjusted	Outcome	Unbalanced	2,638	1.14	1.2	0	1.9	0	11.72	1.5
			Balanced	2,535	1.14	1.2	0	1.9	0	11.72	
		Volume	Unbalanced	2,638	216.1	192	103	277	68	2179	
			Balanced	2,535	214.8	190.8	103	276	68	2179	

Indicator	Indicator description (short)	Variable	Data set	n (data points)	$\bar{x}$	s	$x_{0.2}$	$x_{0.8}$	min	max	Rate[1] (%)
STROKE	Stroke; SMR; risk-adjusted	Outcome	Unbalanced	9,893	1.1	0.46	0.76	1.42	0	4.02	14.42
			Balanced	8,745	1.09	0.45	0.76	1.41	0	3.9	
		Volume	Unbalanced	9,893	99.8	91.1	26	167	7	680	
			Balanced	8,745	92.6	92.6	28	173	7	680	
AMI	Acute myocardial infarction (AMI); SMR; risk-adjusted	Outcome	Unbalanced	10,492	1.15	0.5	0.77	1.48	0	5.1	14.85
			Balanced	9,262	1.15	0.49	0.78	1.47	0	4.84	
		Volume	Unbalanced	10,492	68.8	61.3	22	109	7	464	
			Balanced	9,262	71.6	61.9	23	112	7	464	

Note:
The number of examined data points and the arithmetic mean, standard deviation, 20%-quantile, 80%-quantile, minimum and maximum value of the variables outcome and volume are shown for the balanced and unbalanced data set of each indicator.
[1] Rate is the observed frequency of events in the reference population (all patients in this treatment area in Germany) per year. It is independent of the variables and data sets and therefore applies to the indicator itself.
