# Supplementary material for: Stability of hospital quality indicators over time: A multi-year observational study of German hospital data
Source: PLoS One. 2023 Nov 7;18(11):e0293723. doi: 10.1371/journal.pone.0293723 (PMC10629650; doi:10.1371/journal.pone.0293723)
Supplement: S3 Appendix — (PDF) [file pone.0293723.s003.pdf]

APPENDIX 3: *Characteristics of the included hospitals per indicator and data set (balanced and unbalanced)*

Indicator	Indicator description (short)	Data set	n (# of included hospitals)	n (# of excl. hospitals due to MCV*)	Average # of beds	Degree of urbanisation[1] (in %)				Hospital operator type[2] (in %)			Teach. hosp. (%)	Univ. hosp. (%)
						1	2	3	4	1	2	3		
PNEU	Community acquired pneumonia; SMR; risk-adjusted	Unbalanced	1,105	131	320.1	20	28	20	31	35	37	28	48	2
		Balanced	875		347.9	20	29	20	31	35	39	26	53	3
DECU	Decubitus ulcer; ratio of ulcers acquired in hospital; risk-adjusted	Unbalanced	1,451	184	278.9	21	29	21	30	37	43	20	58	3
		Balanced	1,152		313.2	22	28	20	30	37	44	19	60	3
CHOLEC	Cholecystecomy; ratio or reinterventions; risk-adjusted	Unbalanced	1,047	108	360.1	18	30	18	33	41	41	18	60	4
		Balanced	979		360.1	18	30	18	33	41	40	18	59	4
HIPFR	Hip fracture repair; SMR; risk-adjusted	Unbalanced	1,055	130	355.5	18	29	18	35	40	42	18	59	4
		Balanced	991		356.6	19	29	18	34	40	41	19	59	4
HIPREPDI	Hip replacement; ratio of implant dislocations; risk-adjusted	Unbalanced	717	491	395.4	15	32	18	35	37	41	21	62	5
		Balanced	664		393.1	16	32	18	35	37	41	23	62	5
HIPREPRE	Hip replacement; ratio of reoperations; risk-adjusted	Unbalanced	891	251	374	17	31	18	34	39	40	21	59	4
		Balanced	845		375.3	17	31	18	34	39	39	22	60	4
STROKE[3]	Stroke; SMR; risk-adjusted	Unbalanced	1,028	671	496.5	16	20	17	28	36	45	19	62	5
		Balanced	795		489.8	20	24	21	32	36	44	20	63	5
AMI[3]	Acute myocardial infarction (AMI); SMR; risk-adjusted	Unbalanced	1,082	499	493	17	15	23	16	41	42	17	64	5
		Balanced	842		491	18	28	19	33	41	41	18	65	5

Notes: * Minimum case volume per hospital (see appendix table 6) ** Teaching hospitals *** University hospitals

[1] *Degree of urbanisation according to the classification of the Federal Institute for Research on Building, Urban Affairs and Spatial Development (BBSR) (categories): 1 cities (densely populated areas), 2 and 3 towns and suburbs (intermediate density areas), 4 rural areas (thinly populated areas)*

[2] *Hospital operator type (categories): 1 non-profit private, 2 non-profit public, 3 private*

[3] *In indicators STROKE (ID 2002) and AMI (ID 2001), individual hospital sites are not considered separately*
