# Supplementary material for: Stability of hospital quality indicators over time: A multi-year observational study of German hospital data
Source: PLoS One. 2023 Nov 7;18(11):e0293723. doi: 10.1371/journal.pone.0293723 (PMC10629650; doi:10.1371/journal.pone.0293723)
Supplement: S4 Appendix — (PDF) [file pone.0293723.s004.pdf]

APPENDIX 4: *Indicator-specific minimum case volume and case volume categories (Cat.) per quality indicator*

**Indicator**	**Indicator description (short)**	**Minimum case volume / Cat. 1**	**Cat. 2**	**Cat. 3**
		Case volume greater than…		
PNEU	Community acquired pneumonia; SMR; risk-adjusted	8	104	199
DECU	Decubitus ulcer; ratio of ulcers acquired in hospital; risk-adjusted	268	4,903	10,822
HIPREPDI	Hip replacement; ratio of implant dislocations; risk-adjusted	102	152	230
HIPREPRE	Hip replacement; ratio of reoperations; risk-adjusted	68	127	203
HIPFR	Hip fracture repair; SMR; risk-adjusted	21	76	119
CHOLEC	Cholecystecomy; ratio or reinterventions; risk-adjusted	41	127	194
STROKE	Stroke; SMR; risk-adjusted	7	25	83
AMI	Acute myocardial infarction (AMI); SMR; risk-adjusted	7	25	67

Notes:
The minimum case volume represents the minimum number of cases that a hospital had to fulfill on average in the survey period in order to be included in the analysis. The hospitals below this volume were excluded. The volume categories (Cat.) shown were used in the GEE (figure 2).
